# CVD phenotyping in oncologic disorders: cardio-miRNAs as a potential target to improve individual outcomes in revers cardio-oncology

**DOI:** 10.1186/s12967-023-04680-9

**Published:** 2024-01-12

**Authors:** Ming Yang, Tiepeng Li, Shujin Guo, Kangping Song, Chuhui Gong, Ning Huang, Dejiang Pang, Hengyi Xiao

**Affiliations:** 1 School of Basic Medical Sciences, Chengdu University of Traditional Chinese Medicine, Chengdu, China; 2 The Lab of Aging Research, State Key Laboratory of Biotherapy, National Clinical Research Center for Geriatrics, West China Hospital, Sichuan University, Chengdu, China; 3 Department of Health Management & Institute of Health Management, Sichuan Provincial People’s Hospital, University of Electronic Science and Technology of China, Chengdu, China; 4 Rehabilitation Medicine Center, West China Hospital, Sichuan University, Chengdu, China; 5 Department of Neurology, Laboratory of Neurodegenerative Disorders, National Clinical Research Center for Geriatric, West China Hospital, Sichuan University, Chengdu, China

**Keywords:** Phenotype of CVD and cancer, Common risk factors, Reverse cardio-oncology, Remote crosstalk and the link, The systemic and holistic characteristics, Cardio-miRNAs, Circulation, Precision medicine, Protecting cardiovascular function

## Abstract

With the increase of aging population and prevalence of obesity, the incidence of cardiovascular disease (CVD) and cancer has also presented an increasing tendency. These two different diseases, which share some common risk factors. Relevant studies in the field of reversing Cardio-Oncology have shown that the phenotype of CVD has a significant adverse effect on tumor prognosis, which is mainly manifested by a positive correlation between CVD and malignant progression of concomitant tumors. This distal crosstalk and the link between different diseases makes us aware of the importance of diagnosis, prediction, management and personalized treatment of systemic diseases. The circulatory system bridges the interaction between CVD and cancer, which suggests that we need to fully consider the systemic and holistic characteristics of these two diseases in the process of clinical treatment. The circulating exosome-miRNAs has been intrinsically associated with CVD -related regulation, which has become one of the focuses on clinical and basic research (as biomarker). The changes in the expression profiles of cardiovascular disease-associated miRNAs (Cardio-miRNAs) may adversely affect concomitant tumors. In this article, we sorted and screened CVD and tumor-related miRNA data based on literature, then summarized their commonalities and characteristics (several important pathways), and further discussed the conclusions of Cardio-Oncology related experimental studies. We take a holistic approach to considering CVD as a risk factor for tumor malignancy, which provides an in-depth analysis of the various regulatory mechanisms or pathways involved in the dual attribute miRNAs (Cardio-/Onco-miRNAs). These mechanisms will be key to revealing the systemic effects of CVD on tumors and highlight the holistic nature of different diseases. Therefore, the Cardio-miRNAs should be given great attention from researchers in the field of CVD and tumors, which might become new targets for tumor treatment. Meanwhile, based on the principles of precision medicine (such as the predictive preventive personalized medicine, 3PM) and reverse Cardio-oncology to better improve individual outcomes, we should consider developing personalized medicine and systemic therapy for cancer from the perspective of protecting cardiovascular function.

## Introduction

Micro-RNA (miRNA), as a non-coding small RNA composed of 18–25 nucleotides, which is one of the main elements involved in intracellular post-transcriptional regulation [1, 2]. MiRNA are mainly derived from the tissue cells, exosomes, microenvironment and body fluids. The exosomal miRNAs are ubiquitous and important factors that have a systemic and holistic impact on the body [3–6]. Therefore, circulating exosomal miRNAs provides them with a condition to involve in the connection and regulation between different diseases. Based on numerous studies, the circulating miRNAs have a significant intrinsic correlation with certain diseases, such as CVD [7], diabetes [8], obesity [9] and tumors [10]. The detection of humoral biomarkers for disease prediction, prevention and personalized treatment is a major development in medicine, so the combination of exosome miRNAs and 3PM can help in the diagnosis of different diseases [11–13]. Therefore, we need to conform the principles of 3PM to consider the overall impact between different diseases based on the perspective of miRNAs [14], which may be better to improve the outcomes of intervention and treatment.

**Fig. 1:**
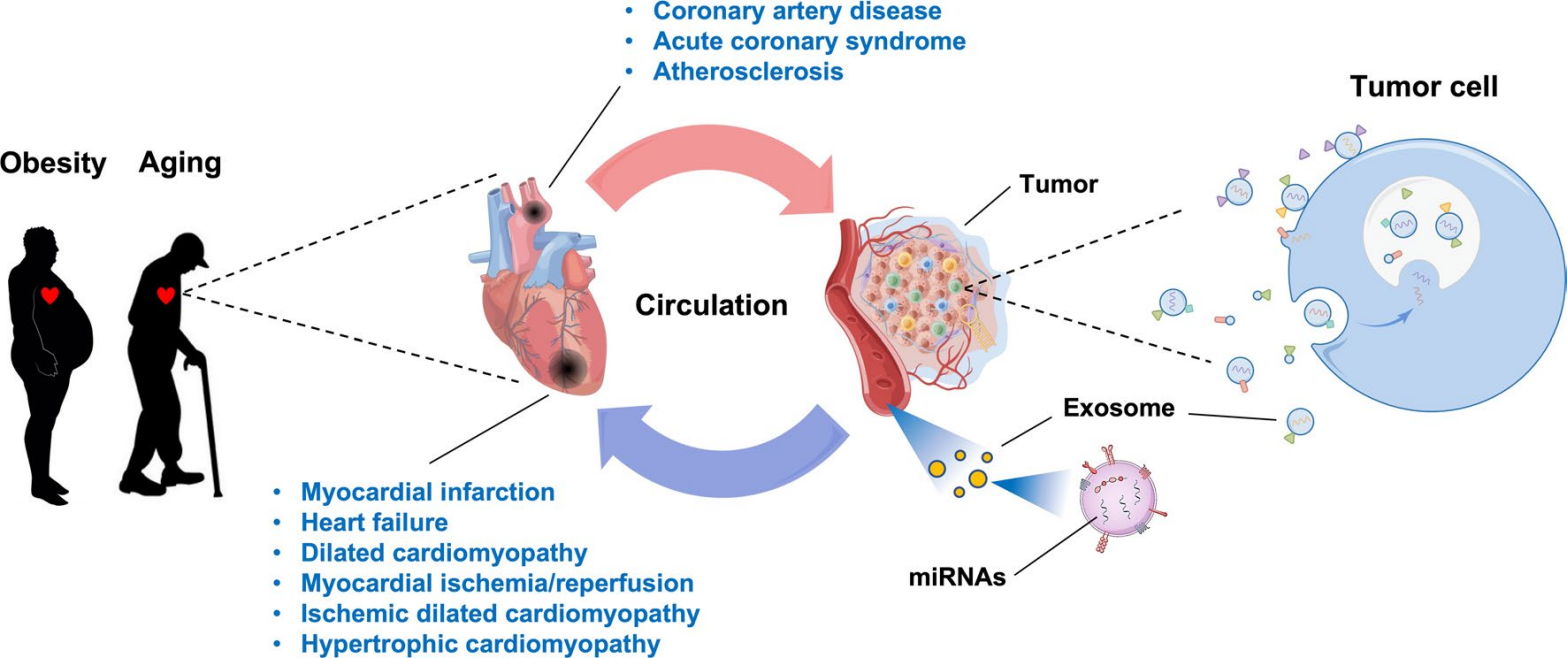
Effects of Cardio-miRNA on tumors via circulation. The Cardio-miRNAs derived from plasma exosomes from obese or aging populations can influence adaptive survival or progression of tumor cells via circulation

CVD-related metabolic dysfunction and chronic stress damage caused by adverse factors (such as high cholesterol, oxidized low-density lipoprotein and hyperglycemia in circulation), which further leads to dysfunction of the circulating compositions [15–18]. Meanwhile, the CVD progression can lead to changes of miRNAs expression profile in the internal environment of blood, thereby affecting the function of other tissues and organs [15, 16]. CVD and tumor are generally considered to be two chronic diseases with the aging process of tissues and organs, both of which could be linked through the blood circulation system and have close material exchange via the peripheral environment for all times [19–22] (Fig. 1). Based on this particularity, the detection of biomarkers is particularly important in the prediction and prevention of systemic diseases. The mechanism of the CVD-related diseases impact on tumors belongs to the field of reverse Cardio-Oncology, which can help us understand and recognize the systemic and holistic effects of CVD on distant cancers via circulation [23–26].

**Fig. 2:**
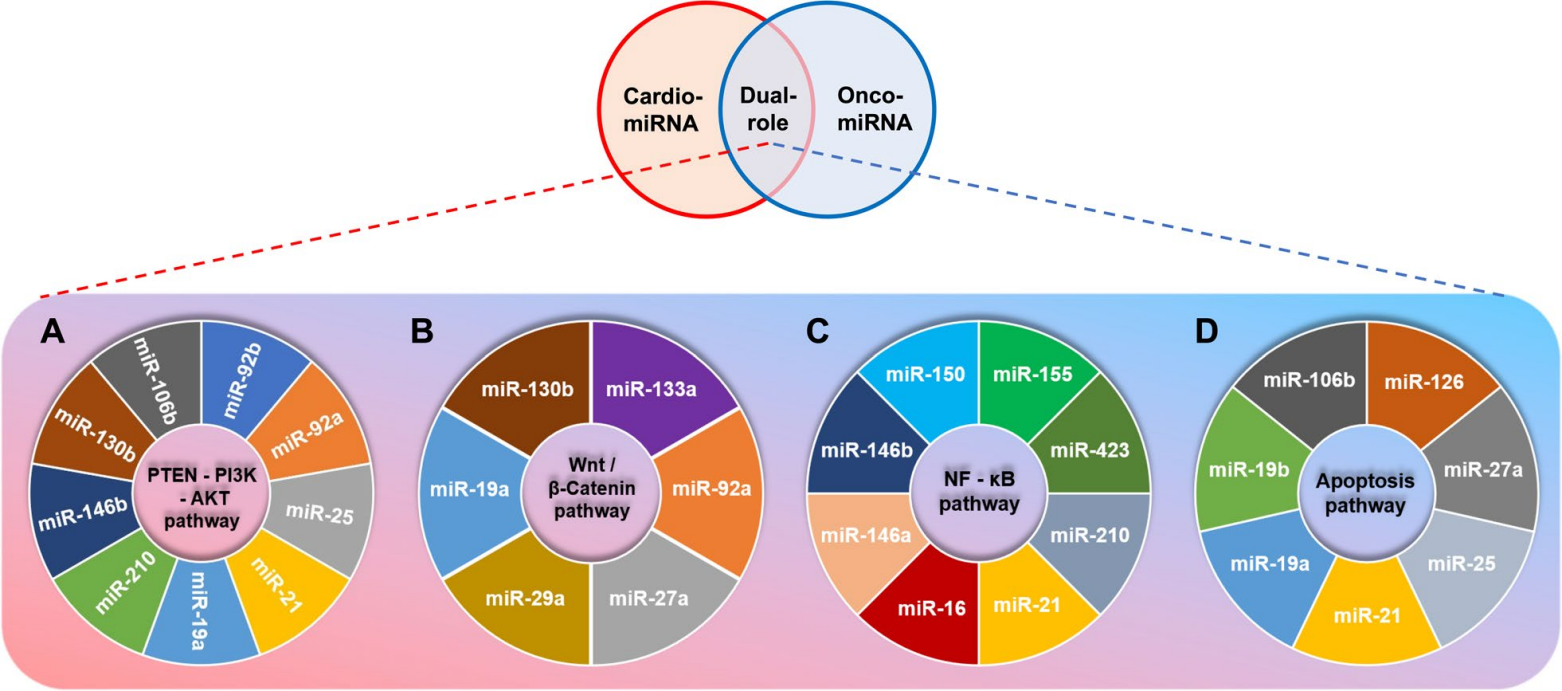
Cardio-/Onco-miRNA is involved in regulating four signaling pathways for adaptive survival of tumor cells. **A** Cardio-miRNAs that regulating the PTEN/PI3K/AKT signaling pathway; **B** cardio-miRNAs that regulating the Wnt/β-Catein signaling pathway; **C** cardio-miRNAs that regulating the NF-κB signaling pathway; **D** cardio-miRNAs that regulating the apoptosis signaling pathway

Tumor is a relatively heterogeneous tissue in the body that is affected by the distribution or density of blood circulation, which is characterized by frequent material exchange with the environment in the blood. For example, the regulation of tumor microenvironment, tumor immune infiltration and metabolic reprogramming can be affected by cardiovascular disease-related miRNAs (Cardio-miRNAs) [27–29]. Meanwhile, the appreciable effects of exosomes-miRNAs on tumor lesions via the circulatory system is a necessary condition for the adaptive survival and progression of tumor cells [29]. Based on these facts, Cardio-miRNA may adversely affect the treatment of tumors and act as a factor to promote tumor miRNAs (Onco-miRNAs), which may lead to poor prognosis of patients with concomitant tumors [30] (Fig. 2 and Table 1).

**Table 1:** Regulation of tumor malignancy corresponding to Cardio-miRNAs

Cardiovascular disease (CVD)-miRNAs					Tumor-promoting effect		
miRNA	Source^a^	Expression in Change	Pathology	References	Tumor	Target in tumor tissue	References
miR-16	Circulation	↑,↓	ICM, AS, CAM	[101, 115]	LUAD, HCC NSCLC	TFAP2A/PSG9/TGF-β LDH-A/lactate/NF-κB	[131, 160, 184]
miR-19a	Circulation	↑	AMI, CAD	[90, 91]	GC, HCC GC UC ccRCC	PTEN/PI3K/AKT SMAD2/Wnt/β-catenin Bim—apoptosis PTEN/SMAD4	[54–57, 103, 152]
miR-19b	Circulation	↑	HF, AMI, AS	[142–144]	NSCLC CRC	BCL2L11/PPP2R5E SMAD4, Bim—apoptosis	[103, 152, 153, 185]
miR-21	Circulation	↑	ASO, CAD, HF, AMI	[97, 116, 117, 186]	UC, HCC, NSCLC, CRC, GBM, ccRCC CRC RCC PC	PTEN/PI3K/AKT PTEN/Akt/IKKβ PTEN/Akt/NF-ĸB p53/p21-cyclin E2-Bax/caspase-3 FASL	[57–62, 134, 154, 156, 187]
miR-25	Circulation	↑	HF	[188, 189]	TNBC, RBM, BC, HCC CCA	BTG2/AKT/ERK/MAPK PTEN/AKT, MEK4/JNK1 TRAIL	[63, 64, 77, 155, 190]
miR-27a	Circulation	↑	CAD, HF, AMI	[92–94]	RCC NSCLC GC TNBC	TXNIP SMAD2/SMAD4 PHLPP2/AKT GSK-3β/Wnt/β-catenin	[76, 104, 161, 191]
miR-29a	Circulation	↑	HCM, CHD	[95, 96]	AB	CTNNBIP1/Wnt/β-catenin	[192]
miR-30d	Circulation	↑	HF, AMI	[94, 193]	PC	MYPT1/c-JUN/VEGFA	[168]
miR-92a	Circulation	↑	CAD, CAV	[97, 98]	HCC, NSCLC SCCOT OC CRC	PTEN/PI3K/AKT DKK1/ Wnt/β-catenin PTEN	[65, 66, 105, 194]
miR-92b	Circulation	↑	HF, CAD, PH	[195–197]	GC SCLC, GBM	DAB2IP/PI3K/AKT PTEN/AKT	[67, 78, 198]
miR-106b	Circulation	↑	CAD, HF	[145, 146]	BC, CRC HCC CRC ccRCC	PTEN/PI3K/AKT GPM6A/DYNC1I1/AKT/ERK p21 TRIM8/p21	[68, 69, 82, 158, 199]
miR-126	Circulation	↓,↑	AMI, AS, IHD, SA, UA	[126, 147–151]	ccRCC BALL	SLC7A5/mTOR-HIF p53	[157, 200, 201]
miR-130b	Circulation	↑	MI/R, RCVS	[99, 100]	BC, OS, RCC ES ccRCC NSCLC	PTEN, PTEN/AKT ARHGAP1/CDC42/PAK1/AP1 WNK, TCF4 PTEN/Wnt-β-catenin	[70–73, 169, 202]
miR-133a	Circulation	↓,↑	CAD, ACS, AMI	[102, 186]	OS OC	Bcl-xL/Mcl-1 PYGB/Wnt-β-catenin	[106, 159]
miR-146a	Circulation	↑,↓	AMI, ACS, HF, CAD	[94, 118–120]	NSCLC	TRAF6/NF-ĸB/p65	[135]
miR-146b	Circulation	↑	MI, PH, HF	[121, 122]	TC BC ccRCC	PTEN/PI3K/AKT AUF1/ETS2/MMP2 TRIM2, TRAF6	[74, 79, 80, 203]
miR-150	Circulation	↓,↑	PH, HF, AMI	[123–125]	NSCLC	FOXO4/NF-ĸB/Snail	[132]
miR-155	Circulation	↑	AS, DCM	[126, 127]	MM ccRCC BC CRC	SOCS1/JAK2/STAT3 IGF1R/PI3K/AKT RKIP PPP2CA/AKT/NF-κB	[81, 134, 204, 205]
miR-208	Circulation	↑	CAD, ACS, AMI	[186, 206, 207]	PC HCC	E-Cadherin/PI3K/AKT/ GSK-3β ARID2/IFITM1	[163, 208]
miR-200a	Circulation	↑	HCM, SCA	[209, 210]	BC	Dicer/miR-16/JNK2/MMP-2 axis	[211]
miR-210	Circulation	↑	PH, HF, CAD	[91, 129, 130]	PC NSCLC OC	OCS1/TNIP1/p65/NF-κB UPF1/PTEN/PI3K/AKT EphrinA3-PI3K/AKT	[75, 212, 213]
miR-223	Circulation	↑	AS, AMI, SC, AD	[143, 214, 215]	ccRCC	SLC4A4, HIF-2α	[216, 217]
miR-423	Circulation	↑	AMI, HF	[94, 128]	GBM PC BC	ING-4/AKT/ERK GREM2/TGF-β, CREBZF TNIP2/NF-ĸB	[133, 162, 166, 167]
miR-451	Circulation	↓	PH, AMI	[143, 218]	ccRCC	PSMB8	[219, 220]

^a^Circulation refers to clinical samples derived from serum or plasma

The cellular regulatory processes and mechanisms between miRNAs and CVD have been well studied, but the role of miRNAs as a systemic influence in the synthesis of cross-talk between different diseases is still less. Especially, some typical biomarkers reflected by changes of expression profile for Cardio-miRNAs in circulation. We sorted out the relevant mechanism of Cardio/Onco-miRNAs involved in CVD phenotype on tumor regulation according to the objective background of reverse Cardio-Oncology. These mechanisms will be key to revealing the systemic or holistic effects of CVD on tumors, which is also an important value for the application of precision medicine in the diagnosis and treatment of systemic diseases. These mechanisms might serve as evidence to supplement the importance of predictable diagnosis and personalized treatment between CVD and tumors, and it also provides a reference for developing systemic principles of improve individual outcomes.

## The outness of cardio-miRNAs affecting tumor progression via crosstalk

Clinical or basic studies associated with reverse Cardio-Oncology have shown that CVD (e.g. hypertension, heart failure (HF), arteriosclerosis (AS) and myocardial infarction (MI) etc.) can influence distant tumor development by secreting circulating factors, such as the noncoding RNA, cytokines and proteins [23, 26, 31]. The Cardio-miRNAs of cardiovascular dysfunction (e.g., pro-inflammatory and cellular senescence) is an important inducing factor for cardiovascular diseases. [32–34] In addition to heart, the function and key role of blood vessels is manifested by vascular endothelial cells [35–37]. Since the blood vessels are structures that interact directly with tumors, the vascular endothelial cells are involved in secreting exosome miRNAs when under the condition of the stress of various factors. This mechanism is also an important way for self-regulation of CVD via paracrine [33], which will affects distal disease progression (such as cancer or tumorigenesis) [30, 38]. However, CVD and cancer share many common risk factors and disease mechanisms, and evidence from some clinical studies suggests that CVD is strongly associated with an increased risk of tumorigenesis [such as, colorectal cancer, liver cancer, lung cancer, melanoma, kidney cancer, lymphoma and breast cancer etc. HR (95% CI) > 1.2, *P* < 0.05] [39–43]. We need to focus on the predictive value of clinical detection of circulating factors in CVD and tumor progression, then develop effective tumor treatment based on the perspective of protecting cardiovascular function.

Evidence from experiments have shown that HF can promote the malignant progression of distal colorectal cancer via circulation [44]. In addition, studies have confirmed that miRNA are important mediators of CVD affecting distant tumor progression from the circulation system [45, 46]. Ye Yuan et al. found that exosome miR-22-3p secreted by cardiomyocytes after myocardial infarction (MI) can promote the malignant progression of distal lung cancer and osteosarcoma, and the main mechanism is the tolerance of tumor cells to ferroptosis [45]. According to these effects of CVD phenotype on tumor, when tumor cells acquire the systemic regulation of relevant signaling pathway from circulating exosome Cardio-miRNAs, it can impact on the malignant process of tumors (Fig. 1) [28]. Therefore, clarifying the physiological regulatory mechanism of tumor cells involved in Cardio-miRNAs is an important basis for the treatment of CVD-associated tumors.

## The cardio-miRNAs regulate adaptive survival of tumor cells via four major pathways

Based on the reported contribution of related miRNAs to the regulatory mechanism of malignancy process of tumor cells, combining with the fact that exosome-derived cardio-miRNAs can regulate tumors via circulation, we have deeply analyzed and summarized the different signaling pathways involved in Cardio-miRNAs. The aim is to predict and evaluate the impact on adaptive survival of tumor cells based on the regulatory mechanisms of different pathways and related Cardio-miRNAs as markers. Different expression levels of circulating cardio-miRNAs predict the progression of concomitant tumors and can be used to develop personalized treatment options.

### PTEN/PI3K/AKT pathway in tumor

PTEN (Phosphatase and Tensin Homolog), as a tumor suppressor, which has been found inactive in different types of tumor cells. Therefore, the function of PTEN has considered to be one of the important factors affecting the human tumorigenesis [47]. In normal cells, the tumor inhibitory effect of PTEN is mainly achieved by inhibiting the activity of the PI3K/AKT signaling pathway [48]. Meanwhile, the PTEN/PI3K/AKT pathway is involved in regulating important pathways for tumor cell cycle, proliferation and malignant progression, and the expression level and functional activity of PTEN are key to the adaptive survival of tumor cells [49]. Various non-coding RNAs, such as miRNA, lncRNA and cirRNA. etc., have been reported to be involved in the post-transcriptional regulation of PTEN [48]. Exosomes, as a main carrier for miRNAs, is one of the main ways in which the external environment affects tumor cells [50]. MiRNAs affect the function of various organs in the body via blood circulation [4, 51, 52]. Aging and obesity are major factors contributing to the prevalence and high incidence of cardiovascular disease in the population [9, 53]. Therefore, the upregulation of Cardio-miRNAs from circulation may become an important factor affecting middle-aged and elderly or obese tumor patients [30].

**Fig. 3:**
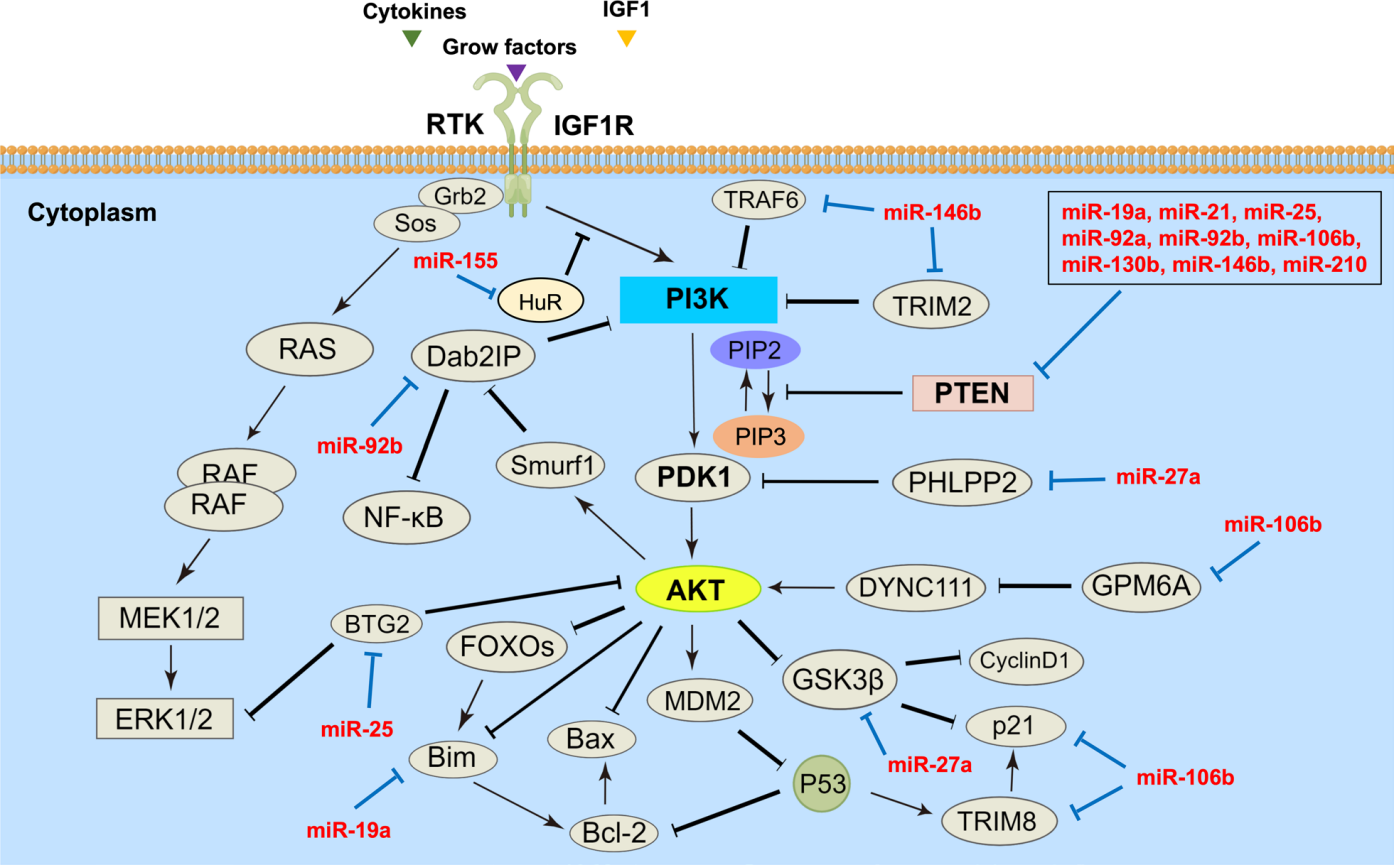
Cardio-miRNAs regulate tumor cells adaptive progression through the PTEN/PI3K/AKT signaling pathway. Bold black indicates key node protein factors involved in the pathway; Bold red letters indicate Cardio-miRNAs. (The red bold represents circulation-derived exosomes Cardio-miRNAs, which may be upregulated in tumor cells; solid arrows indicate promotion or activation; Line segment indicate inhibition)

Based on the results of the clinical research and public database (TCGA), we have summarized 9 Cardio-miRNAs that are reported targeting at PTEN (Fig. 2A, Table 1), such as miR-19a (GC, HCC, RCC) [54–56], miR-21 (UC, HCC, NSCLC, CRC, GBM, ccRCC, RCC, PC) [57–62], miR-25 (BC, HCC) [63, 64], miR-92a/b (HCC, OC, GBM) [65–67], miR-106b (BC, CRC) [68, 69], miR-130b (BC, NSCLC, OS, RCC) [70–73], miR-146b (TC) [74], miR-210 (NSCLC) [75] (Fig. 3). In tumor cells, these miRNAs attenuate the inhibitory effect on the PI3K–AKT signaling pathway by directly targeting PTEN. Therefore, PTEN can be regarded as multiple circulating Cardio-miRNA targets and has a wide range of effects on the development of different tumor types, which reflects the role in cross-disease linkage of cardio-miRNAs.

In addition, there are several other miRNAs indirectly involved in regulating and activating PI3K–AKT signaling axis. For example, miR-27a, by targeting PHLPP2, attenuates the inhibition of PDK1/AKT pathway, thereby promoting the malignancy of GC cells [76]. Similarly, miR-25 attenuates the inhibitory effect on the BTG2/AKT pathway and promotes the proliferation of TNBC cells by targeting BTG2 [77]. In GC cells, miR-92b indirectly attenuates the activation inhibition of PI3K–AKT signaling pathway by targeting Dab2IP, and ultimately promotes tumor progression [78]. MiR146b attenuates the activity inhibition of the PI3K–AKT pathway by targeting TRAF6 and TRIM2, respectively, and promotes the course of RCC patients [79, 80]. The M2 macrophage-derived exosome miR-155 eliminates transduction inhibition of IGF1R by targeting HuR (Human antigen R), after which activating the PI3K–AKT pathway and promoting the development of ccRCC [81]. MiR-106b from hepatoma cells activates the AKT/ERK pathway via targeting GPM6A, resulting in upregulation of DYNC1I1 expression and ultimately promoting HCC cell proliferation [82]. In summary, among the various Cardio-miRNAs associated with the PTEN/PI3K/AKT pathway, the one that directly target PTEN may be important factors resulting in the poor prognosis of concomitant tumors.

### Wnt/β-catenin pathway in tumor

Wnt is a ligand protein containing 19 glycoprotein families in mammalian cells [83], which is involved in regulating cell proliferation, adhesion, migration and differentiation through β-catenin-dependent or non-dependent forms [84]. Based on the proliferation pattern and adaptive survival phenotype of tumor cells, it also reflects the abnormal regulatory mechanism of the Wnt/β-Catenin pathway [84]. β-catenin is an adaptor protein that coordinates signal transduction in the Wnt signaling pathway, and it is capable of nuclear translocation to participate in the transcription of EMT-related genes in tumor cells [85]. There are important noncoding RNAs for post-transcriptional regulation of Wnt/β-Catenin pathway, among which miRNAs are ones that involved in regulating malignant progression or adaptive survival of tumors during the Wnt/β-Catenin signal transduction of tumor cells [86–89].

**Fig. 4:**
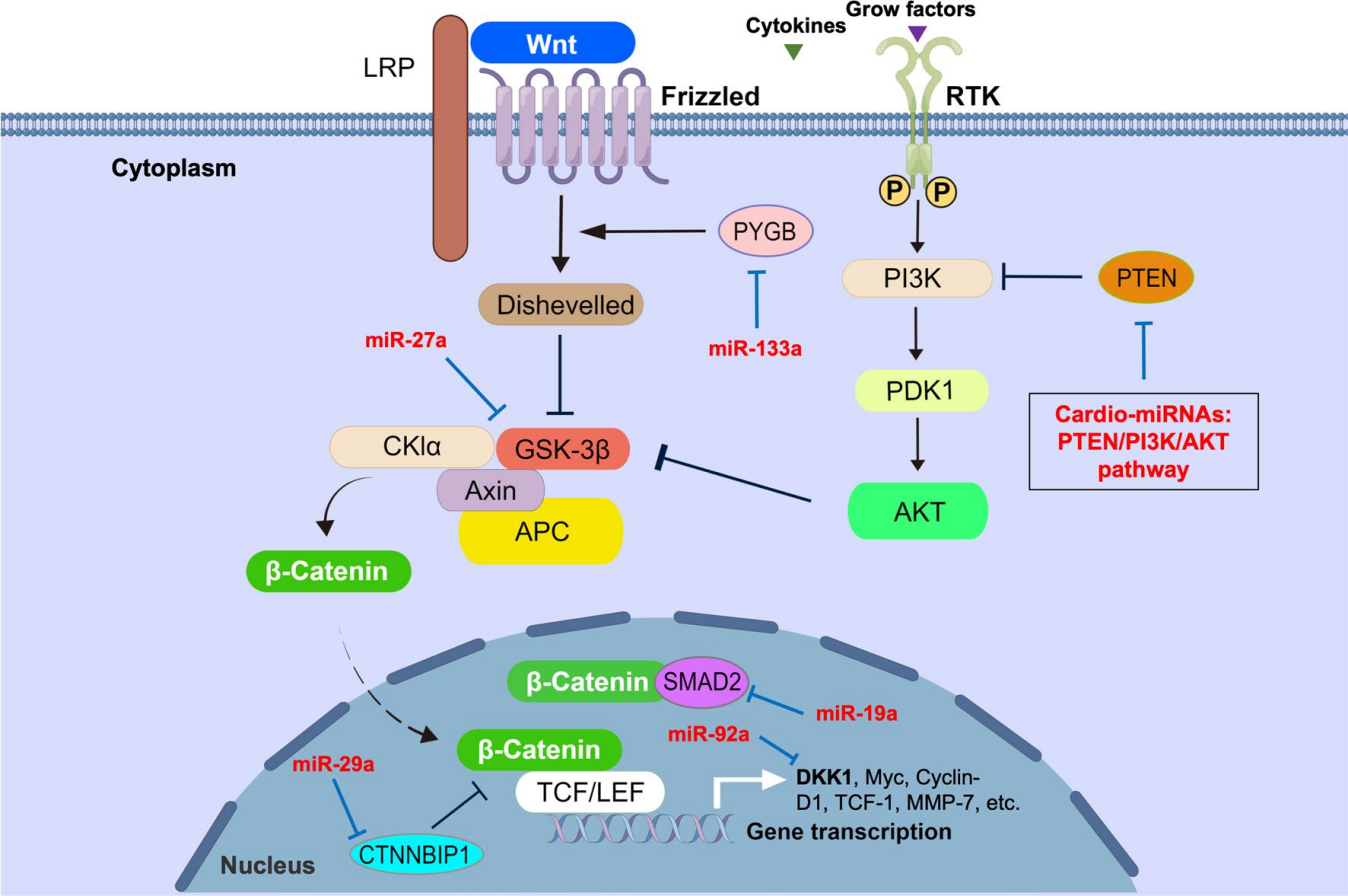
Cardio-miRNAs regulate tumor cells adaptive progression through the Wnt/β-Catein signaling pathway. Bold white indicates key node protein factors involved in the pathway; Bold red letters indicate Cardio-miRNAs. (The red bold represents circulation-derived exosomes Cardio-miRNAs, which may be upregulated in tumor cells; solid black arrows indicate promotion or activation; Line segment indicate inhibition; dashed arrows indicate multi-step transfers; White corner arrows indicate gene transcription expression)

Among the Cardio-miRNAs, 6 miRNAs were associated with AMI, CAD, HF, HCM, CHD, CAV, MI/R, RCVS and ACS, and were upregulated in circulation (Table 1) [90–102]. These Cardio-miRNAs can promote the progression of tumor cell malignancy by targeting relevant proteins in the Wnt/β-Catenin pathway (Fig. 2B, Table 1). To be specific, miR-19a can affect the competitive binding of SMAD2 to β-Catenin and promote the EMT process of GC cells via targeting SMAD2 [103]. MiR-27a can promote the malignant metastasis of TNBC cells by targeting GSK-3β to cause more release of β-Catenin and nuclear transposition [104]. MiR-92a and miR-133a targeted DKK1 and DYGB, respectively, which attenuate the inhibitory effect of these two proteins in the process of Wnt signal transduction, thereby promoting the progression and cell metastasis of OC [105, 106]. MiR-130b can target at down regulating the level of PTEN, following by an attenuation of the inhibition in PI3K/AKT/GSK-3β/β-Catenin pathway, and ultimately resulting in the malignant metastasis of NSCLC [73] (Fig. 4). To summarize, Cardio-miRNAs from circulation are involved in the signal transduction of the Wnt/β-Catenin pathway by targeting other cytokines, thereby promoting malignant metastasis or progression of tumors.

### NF-κB pathway in tumor

The NF-κB pathway is one of the most important pathways involved in the regulation of cell physiology and pathometabolism, which includes inflammatory response, apoptosis, differentiation, immune response and cell migration [107, 108]. However, in most cases, NF-κB pathway is related to regulating cellular pro-inflammatory responses and survival, such as the adaptive survival regulation of tumor cells. This typical mechanism was proved by the association between low-level pro-inflammatory response and energy metabolism in tumor cells [109, 110]. In most tumor cells, the NF-κB pathway is highly activated and it mediates the malignant proliferation or survival of cells via nuclear metastasis, and ultimately promotes the metastasis and angiogenesis of tumor [108, 111–113]. Therefore, the post-transcriptional regulation of NF-κB signaling pathway by tumor cells is a key process for the adaptive survival of tumor cells [113, 114].

**Fig. 5:**
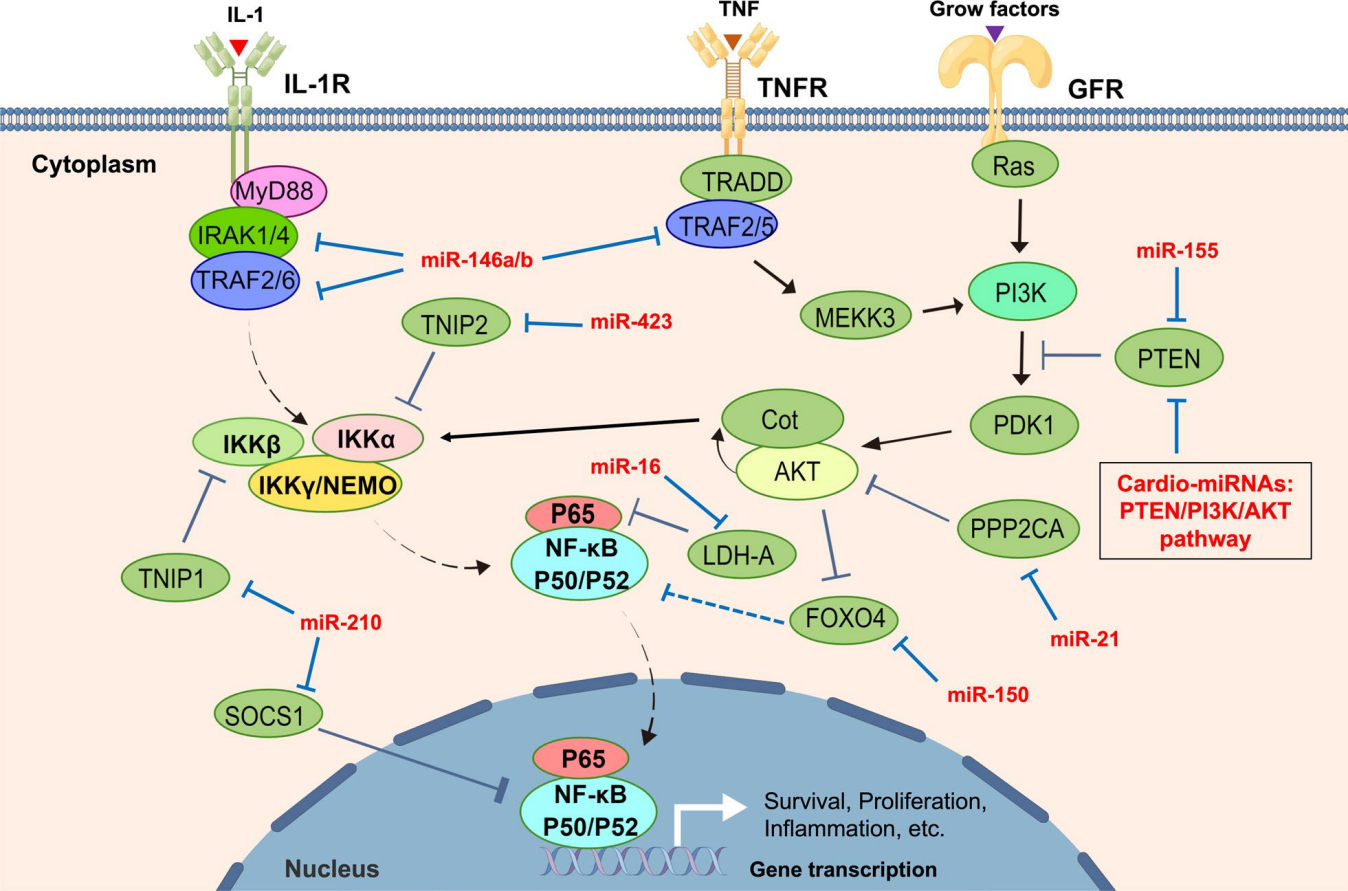
Cardio-miRNAs regulate tumor cells adaptive progression through the NF-κB signaling pathway. Bold black indicates key node protein factors involved in the pathway; bold red letters indicate Cardio-miRNAs. (The red bold represents circulation-derived exosomes Cardio-miRNAs, which may be upregulated in tumor cells; solid black arrows indicate promotion or activation; line segment indicate inhibition; dashed arrows indicate multi-step transfers or activation; white corner arrows indicate gene transcription expression)

Circulation in patients with cardiovascular diseases, such as ICM, CAD, ASO, HF, AMI, ACS, PH, DCM, provides a way for the transmission of inflammatory response [91, 94, 97, 101, 115–130], and the cardio-miRNAs spread through this way may adversely affect the treatment of concomitant tumors during circulating process (Fig. 2C, Table 1). For example, miR-16, miR-150 and miR-423 indirectly activated the NF-κB signaling pathway by targeting LDH-A, FOXO4 and TNIP2, respectively, and promoted the progression of NSCLC and BC [131–133]. MiR-21, as a typical noncoding RNA targeting at PTEN, indirectly activates NF-κB signaling in CRC cells via the PI3K–AKT pathway, and ultimately promotes the proliferation of tumor cells [60]. Therefore, it can be speculated that multiple Cardio-miRNA can target PTEN/PI3K/AKT pathway, which may indirectly activate the NF-κB signaling pathway for multi tumor cell types. In addition, miR-155 promotes tumor progression by targeting PPP2CA and indirectly activating the NF-κB signaling pathway via AKT in CRC cells [134]. In the upstream of the NF-κB pathway, miR-146a activates the NF-κB/p65 axis by targeting ligands IRAK1 and TRAF6, ultimately promotes the proliferation of NSCLC cells [135]. As a bidirectional regulator, miR-210 promotes EMT and cell metastasis of PRAD via indirectly activating the NF-κB pathway after targeting SOCS1 and TNIP1 [130] (Fig. 5). In summary, Cardio-miRNAs that targeting PTEN/PI3K/AKT and NF-κB pathway may be typical Onco-miRNAs that promote the progression of different types of tumors. Thus, these Cardio-miRNAs became the keys to the adaptive survival of tumor cells via the cross-combination of these two pathways.

### Apoptosis pathway in tumor

Apoptosis is also one of the most important ways for cells to regulate self-physiology and metabolism, and the function is to maintain the tissues in a physiological state and to remove damaged cells, such as cells with DNA damage and high oncogenes expression [136–138]. In order to achieve adaptive proliferation or survival, tumor cells need to implement inhibition of various signaling pathways involved in apoptosis [139]. Therefore, compared with normal cells, apoptosis signaling activity is deregulated during tumorigenesis, which can be achieved by miRNAs targeting at genes that take parts in pro-apoptotic pathway [140]. In general, miRNAs involved in apoptosis regulation significantly affect the expression levels of pro/anti-apoptotic genes, such as oncogenes, endoplasmic reticulum (ER) stress, and apoptosis-related genes from mitochondrial extramembrane [141].

**Fig. 6:**
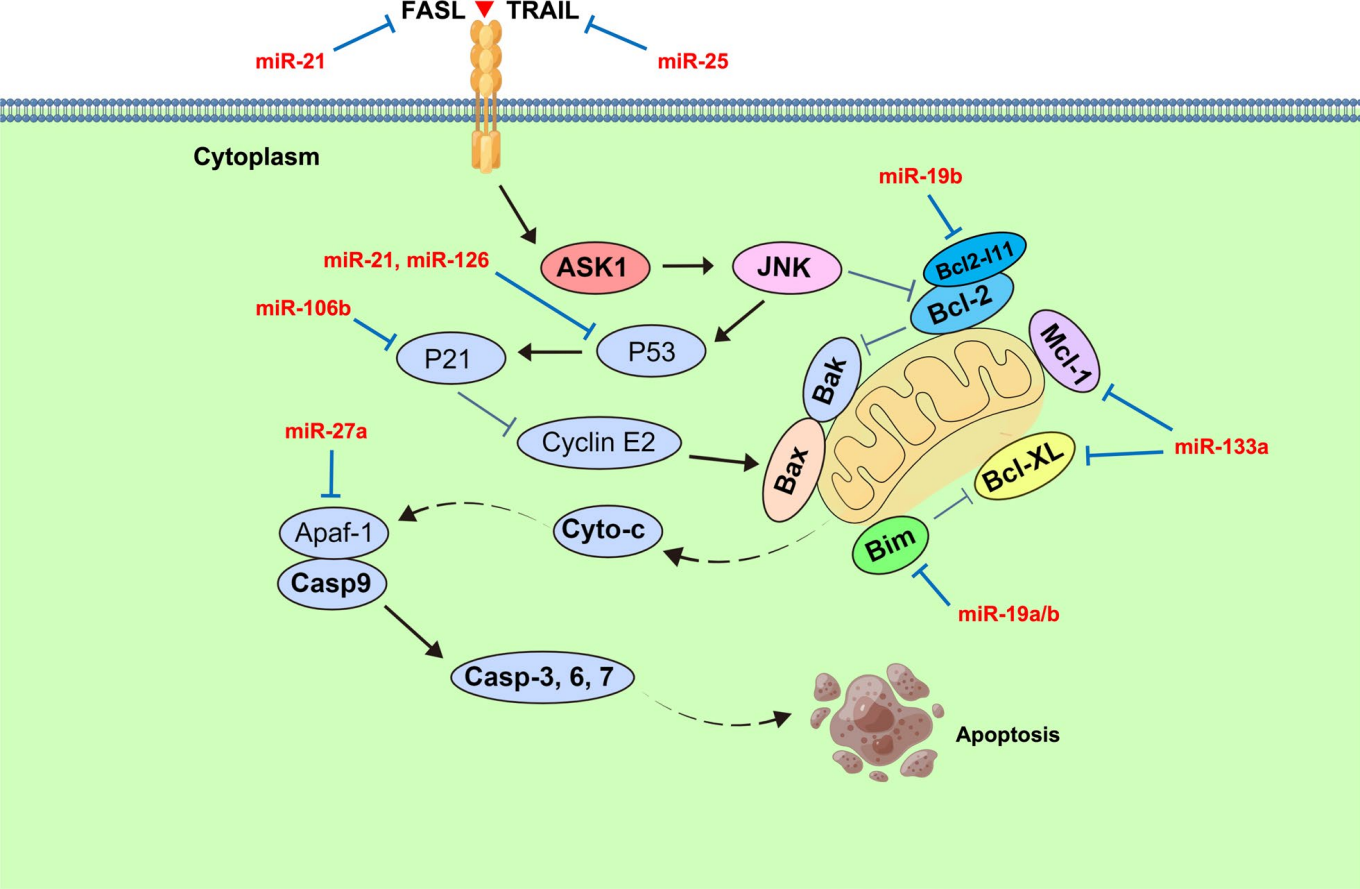
Cardio-miRNAs regulate tumor cells adaptive progression through the apoptosis signaling pathway. Bold black indicates key node protein factors involved in the pathway; Bold red letters indicate Cardio-miRNAs. (The red bold represents circulation-derived exosomes Cardio-miRNAs, which may be upregulated in tumor cells; solid black arrows indicate promotion or activation; Line segment indicate inhibition; Dashed arrows indicate multi-step activation)

Studies have reported that the up or down regulation of some Cardio-miRNAs in circulation can correspondingly target at pro-/ anti-apoptotic proteins [90–94, 97, 101, 116, 117, 126, 142–151] (Fig. 2D, Table 1). For example, miR-19a and miR-19b can target Bim on mitochondria in UC and CRC cells, respectively, and promote tumor cell survival and progression [152, 153]. MiR-21 and miR-25 attenuate the transduction process of apoptosis signaling via targeting exogenous apoptosis-inducing receptors FASL/TRAIL in PRAD and CCA cells, respectively, and ultimately result in chemotherapy resistance of tumor [154, 155]. In addition, miR-21 and miR-126 target p53 in RCC and BALL cells respectively. Besides, miR-106b targeted p21, which ultimately deregulated the activity of p53/p21-cyclinE2-Bax/Casp3 signaling pathway and resulted in chemotherapy resistance or poor prognosis [69, 156–158]. When the expression level of miR-133a is downregulated in circulation, it may attenuate the targeted regulation of Bcl-xL/Mcl-1 and enhance the proliferative activity of OS cells [159] (Fig. 6). To sum up, changes in the expression level of some Cardio-miRNAs may affect the therapeutic effect of patients with concomitant tumors via targeting the genes related to the apoptotic pathway.

### Other cardio-miRNAs in tumor

**Fig. 7:**
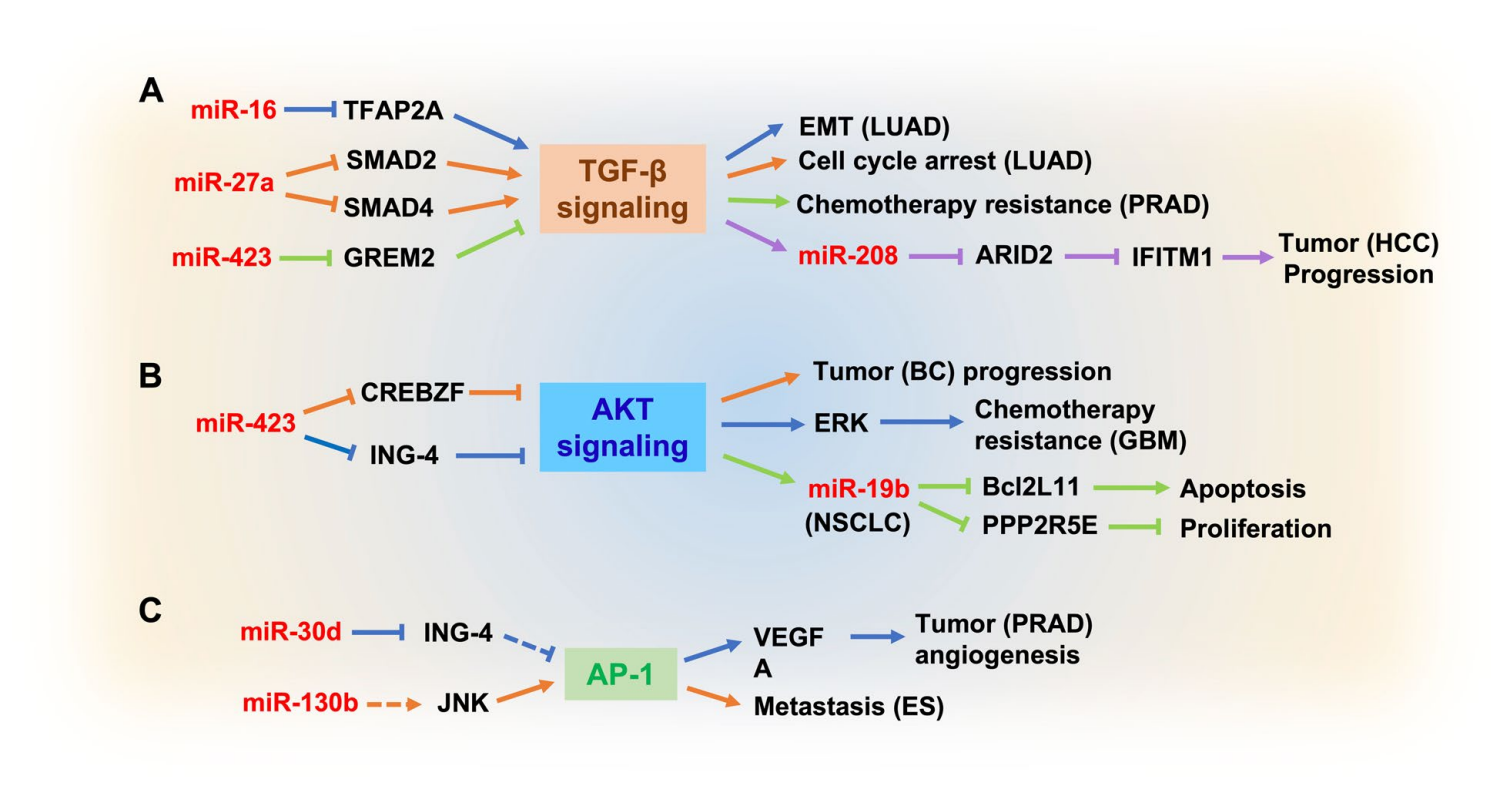
Regulation of tumor cells adaptive progression by other cardio-miRNAs. **A** Cardio-miRNA regulates the TGF-β signaling pathway; **B** cardio-miRNA regulates the AKT signaling pathway; **C** cardio-miRNA regulates the AP-1 signaling pathway. (The red bold represents circulation-derived exosomes Cardio-miRNAs, which may be upregulated in tumor cells; the arrows/segments corresponding to solid/dashed lines of the same color represent the same pathway)

In addition to the 4 typical regulatory pathways mentioned above, there are also some Cardio-miRNAs that indirectly promote the malignancy progression of tumor cells. We mainly take 3 targets from different pathway as examples to demonstrate the regulatory effect of these Cardio-miRNAs on tumors (Fig. 7, Table 1). MiR-16 and miR-27a regulate the activity of the TGF-β signaling pathway by targeting TFAP2A and SMAD2/SMAD4 respectively, which promote tumor EMT and cell cycle [160, 161]. Furthermore, miR-423 attenuates the inhibition of the TGF-β pathway by targeting GREM2 and results in chemotherapy resistance of patients with PRAD [162]. Meanwhile, TGF-β can promote the expression of miR-208 in HCC cells, which attenuates the inhibitory effect on IFITM1 activity by targeting ARID2, and ultimately promote tumor progression [163] (Fig. 7A). Due to its dual properties and pleiotropy for tumor cells, the TGF-β pathway is a potential target that needs precise control. The mutation, deletion, amplification, methylation of TGF-β and changes of miRNA levels have been proved to have significant effects on TGF-β signaling activity for different cancer types. Therefore, post-transcriptional survival regulation of TGF-β mediated cancer pathways, which provides important molecular perspectives for treatment or research [164, 165].

The level of miR-19b is upregulated by the activation of the EGFR/AKT pathway, which inhibits the apoptosis pathway in NSCLC cells via targeting Bcl2L11 and PPP2R5E, thereby promoting the proliferation of tumor cells [153]. MiR-423 indirectly attenuates the inhibition of AKT via targeting ING-4 and CREBZF, and it promotes chemotherapy resistance in GBM and BC respectively [166, 167] (Fig. 7B). MiR-30d and miR-130b attenuate the inhibition of AP-1 via targeting MYDT1 and ARHGAP1 respectively, which promote the progression of PRAD and ES [168, 169] (Fig. 7C). These reported Cardio-miRNAs can indirectly promote the malignant progression of tumors through the cross-targeting of some other pathways, which reflects the diversity of regulatory mechanisms of Cardio-miRNAs. The regulatory mechanisms involved in this minority Cardio-miRNAs only supplement the four major pathways mentioned above. In fact, there are some other regulatory mechanisms that need to be further improved according to the latest studies reports in the future.

## Perspectives and challenges

The cellular regulatory processes and mechanisms between miRNAs and CVD have been well studied [170–172]. However, the role of miRNAs as a systemic influence in the synthesis of crosstalk between different diseases is still less. Especially, some typical biomarkers reflected by changes of expression profile for Cardio-miRNAs in circulation, which may become an important factor that diseases associated with age [26, 30, 173]. Therefore, Cardio-miRNAs may be keys to potential targets that treating chronic complications and malignant progression of tumor [30, 174]. We should also pay more attention to these adverse effects that reverse Cardio-Oncology in the clinical treatment of cancer based on the principles of precision medicine. In this way, a holistic approach to multiple diseases, classification and multi-level diagnosis is carried out to evaluate the regulatory mechanism of reverse cardio-oncology [12–14]. Such as miR-21 is a representation that targets multiple signaling pathways, including PTEN/PI3K/AKT, NF-κB and apoptosis signaling pathways etc., and it also exhibits typical characteristics of Onco-miRNAs (Fig. 2) [175–177]. We should predict and evaluate the possible adverse consequences of miR-21 due to underlying metabolic disease, work life and diet according to the 3PM principle. In addition, it cannot be ignored that the upregulation of Cardio-miRNA expression levels may be as a phenotype of the toxic stress damage of chemotherapy drugs on the cardiovascular system during tumor treatment [173]. Particularly, chemotherapy for middle-aged and elderly tumor patients should reduce cardiovascular damage at the same time, because it may be a disadvantage for tumor treatment [178]. Based on the special phenotypes of CVD, we need take a systematic and holistic approach to consider CVD as an important risk factor for tumor malignancy.

However, the adverse effects of Cardio-miRNAs may be a persistent problem for concomitant tumors of aged and obese patients [99]. In middle-aged and older patients, a systemic treatment (3PM) may be more benefit to improving outcomes for concomitant tumors. For example, statins protect cardiovascular by lowering blood lipids or cholesterol, and a combination of drugs can be taken into consideration to treat tumor patients with arteriosclerosis and coronary heart disease. In addition, cardiotoxic chemotherapy drugs (such as anthracyclines), which are often used in chemotherapy for a variety of clinical tumors, and they can be considered to combined with cardioprotective drugs for tumor treatment, which is more likely to achieve a good prognosis (Table 2) [179–182]. Furthermore, molecular therapy has gradually become an important method for tumor treatment, such as the use of inhibitors (reverse complementary mimics) targeting Cardio-miRNAs to reduce their adverse effects during treatment process. However, experimental studies are needed to ensure its safety and efficacy before it can be applied clinically [183].

**Table 2:** Potential drugs for joint pharmacologic prevention of cardiovascular disease and cancer (Masoudkabir et al. [181])

Drug	Direct target	Indirect targets	Action on CVD	Action on cancer
Statins	HMG-CoAreductase inhibition	• AMPK activation • Inhibition of Cyclines & cycline-dependent kinases • Up-regulation of tumor-suppressors (p53, p27, p21) • Inhibition of PI3K, serineethreonine kinases, NF-κB, and MAPKs signaling pathways	Improving endothelial function Plaque stabilization **↓** Atherosclerosis progression ↓ Myocardial infarction and stroke ↓ Cardiovascular mortality	Tumor-suppressor and anti-cancer role through: ↑ Apoptosis ↓ Proliferation ↓ Invasion ↑ Radiosensitization ↓ DNA damage
ASA	Inhibition of COX1	• AMPK activation?	↓ Myocardial infarction and stroke ↓ Cardiovascular mortality	↓ Cancer incidence ↓ Cancer death
ACEIs/ARBs	ACE inhibition/angiotensin II receptor antagonism	• ↓ VEGF expression • PPAR-γ activation	Improving endothelial function Plaque stabilization ↓ Atherosclerosis progression ↓ Myocardial infarction and stroke ↓ Cardiovascular mortality	↓ Cancer incidence Tumor-suppressor and anti-cancer role through: ↓ DNA damage ↑ Apoptosis ↑ Differentiation ↓ Angiogenesis ↓ Cell growth
Metformin	Unknown	• AMPK activation		↓ Cancer incidence Tumor suppression by regulating cellular proliferation, cell cycle progression and cellular survival
TZDs	PPAR-γ agonism	• AMPK activation • Wnt/β-catenin signaling pathway inhibition • IGF-1 inhibition • Inhibition of leptin gene expression	↓ Coronary and carotid atherosclerosis ↓ Thrombus formation and acute myocardial infarction and stroke ↓ Blood pressure	Tumor suppression through: ↓ Angiogenesis ↑ Apoptosis ↓ Self-renewal of cancer cells ↑ Differentiation

*HMG-CoA-reductase* 3-hydroxy-3-methyl-glutaryl-coenzyme A reductase, *AMPK* Adenosine 50 monophosphate -activated protein kinase, *PI3K* phosphoinositide 3-kinase, *NFekB* nuclear factor kappa-B, *MAPK* mitogen-activated kinases, *CVD* cardiovascular disease, *COX1* cyclooxygenase 1, *ACEIs/ARBs* angiotensin-converting enzyme inhibitors/angiotensin II receptor antagonists, *ACE* angiotensin-converting enzyme, *VEGF* vascular endothelial growth factor, *PPAR-g* peroxisome proliferator-activated receptor-g, *TZDs* thiazolidinediones

## Conclusions

Our review concludes that CVD and tumors can be linked through miRNAs, and these miRNAs may have a dual role (Cardio-/Onco-miRNAs). However, with aging, the dysfunctions of cardiovascular system may appear and changes of systematic phenotypes of circulating miRNAs showed the adverse effects of Cardio-miRNAs for middle-aged and elderly or obese tumor patients. This connection and regulatory mechanism may further demonstrate the necessity and foresight of the 3PM principles between diagnosing and treating for different diseases. Furthermore, the dual properties of Cardio-/Onco-miRNAs suggest that CVD is systemic and holistic problem or risk factor affect distant tumor cells via the circulation, which may be a potential target for treatment and intervention. Therefore, based on the perspective of CVD phenotyping in oncologic disorders, we need a systemic evaluation, prediction and diagnosis of the patients with concomitant tumors, which may provide a reference for avoiding poor prognosis.

## Data Availability

All datasets generated and analyzed during the current study are available from the corresponding author on request.

## Abbreviations

AMI: Acute myocardial infarction
MI: Myocardial infarction
ASO: Atherosclerosis obliterans
CAD: Coronary artery disease
ACS: Acute coronary syndrome
HF: Heart failure
AS: Atherosclerosis
PH: Pulmonary hypertension
DCM: Dilated cardiomyopathy
ACAS: Asymptomatic carotid artery stenosis
MI/R: Myocardial ischemia/reperfusion
RCVS: Reversible cerebral vasoconstriction syndrome
SCAD: Spontaneous coronary artery dissection
ICM: Ischemic dilated cardiomyopathy
HCM: Hypertrophic cardiomyopathy
CHD: Coronary heart disease
CAV: Cardiac allograft vasculopathy
IHD: Ischemic heart disease
SA: Stable angina
UA: Unstable angina
CAM: Cardiomyopathy
LC: Lung cancer
AB: Ameloblastoma
BALL: B cell precursor acute lymphoblastic leukemia
CCA: Cholangiocarcinoma
CRC: Colorectal cancer
PRADC: Prostate cancer
GC: Gastric cancer
UC: Urothelial carcinomas
HCC: Hepatocellular carcinoma
NSCLC: Non-small cell lung cancer
GBM: Glioblastoma
BC: Brest cancer
TNBC: Triple-negative breast cancer
RBM: Retinoblastoma
RCC: Renal cell carcinoma
ccRCC: Clear cell renal cell carcinoma
SCLC: Small cell lung cancer
BC: Bladder cancer
ES: Ewing sarcoma
OS: Osteosarcoma
OC: Ovarian cancer
MM: Malignant melanoma
TC: Thyroid cancer
PC: Pancreatic cancer
HCM: Hypertrophic cardiomyopathy
